# Gate-tuned anomalous Hall effect driven by Rashba splitting in intermixed LaAlO_3_/GdTiO_3_/SrTiO_3_

**DOI:** 10.1038/s41598-021-89767-3

**Published:** 2021-05-21

**Authors:** N. Lebedev, M. Stehno, A. Rana, P. Reith, N. Gauquelin, J. Verbeeck, H. Hilgenkamp, A. Brinkman, J. Aarts

**Affiliations:** 1 Kamerlingh Onnes Laboratory, Leiden University, P.O. Box 9504, 2300 RA Leiden, The Netherlands; 2 Physikalisches Institut (EP 3), Universität Würzburg, Am Hubland, 97074 Würzburg, Germany; 3 Center for Advanced Materials and Devices, BML Munjal University (Hero Group), Gurgaon, 122413 India; 4 MESA+ Institute for Nanotechnology, University of Twente, P.O. Box 217, 7500 AE Enschede, The Netherlands; 5 Electron Microscopy for Materials Science, University of Antwerp, Campus Groenenborger Groenenborgerlaan 171, 2020 Antwerpen, Belgium

**Keywords:** Materials science, Surfaces, interfaces and thin films

## Abstract

The Anomalous Hall Effect (AHE) is an important quantity in determining the properties and understanding the behaviour of the two-dimensional electron system forming at the interface of SrTiO_3_-based oxide heterostructures. The occurrence of AHE is often interpreted as a signature of ferromagnetism, but it is becoming more and more clear that also paramagnets may contribute to AHE. We studied the influence of magnetic ions by measuring intermixed LaAlO_3_/GdTiO_3_/SrTiO_3_ at temperatures below 10 K. We find that, as function of gate voltage, the system undergoes a Lifshitz transition while at the same time an onset of AHE is observed. However, we do not observe clear signs of ferromagnetism. We argue the AHE to be due to the change in Rashba spin-orbit coupling at the Lifshitz transition and conclude that also paramagnetic moments which are easily polarizable at low temperatures and high magnetic fields lead to the presence of AHE, which needs to be taken into account when extracting carrier densities and mobilities.

## Introduction

The two-dimensional electron system (2DES) which is present at SrTiO_3_-based oxide interfaces is of interest as a model system for the physics of band formation and electrical transport in a quantum well where 3*d* electrons are the carriers. Moreover, the system has built-in electrical tunability, since the high permittivity of the SrTiO_3_ substrate allows it to be used as a back gate, thereby varying the shape of the well, the number of carriers, and the population of the various 3*d* subbands. One of the still outstanding questions is whether and how the 2DES can be used as a platform for spintronics, meaning that the electron system can be (tunably) magnetically polarized and furnish not only charge current but also spin currents. The occurrence of Rashba-type spin-orbit coupling (SOC) at the interface is of obvious importance here, and it should be noted that the effect of back-gating does not only change the carrier concentration at the interface, but also changes the strength of the SOC^1,2^. This is a somewhat subtle band structure effect in which the system switches from one- to two-carrier transport at the so-called Lifshitz point^3^. That leads to a strong increase in SOC, and to a change in coupling between itinerant electrons and localized moments. Recently, this effect was utilized for spin-to-charge conversion by injecting spin-polarized carriers^4,5^.

At the archetypical interface LaAlO_3_/SrTiO_3_ (LAO/STO), the occurrence of magnetism is still a much-debated subject, mainly because the nominal ions at the interface (including Ti$^{4+}$) are non-magnetic and defect physics appears to be at its origin. Using magnetic ions seems to be a more controlled route. Magnetic 4*f* ions can easily be substituted for La, while using titanates rather than LaAlO_3_ yields (magnetic) Ti$^{3+}$ ions. In this spirit, delta doping was lately used as a tool to enhance the number of magnetic ions at the interface. In different studies, ultrathin layers of EuTiO$_3$ (ETO)^6^ and (La,Sr)MnO$_3$ (LSMO)^7^ were sandwiched between STO and LAO. Alternatively, La(Al,Mn)O$_3$ (LAMO; up to 30% Mn) was grown on STO in order to bring magnetic ions closer to the interface^8^. In all cases, ferromagnetism was reported, as well as tunability of the effect with a gate voltage.

In our work we concentrate on two questions. One is the question of the mechanism for tunability. We will show in particular that crossing the Lifshitz point by gating can lead to the onset of the AHE. The other is whether the observation of the AHE can be used to infer the presence of magnetic Long Range Order (LRO). Guided by observations in a very different 2DEG, MgZnO/MgO^9^, we conclude that the presence of highly polarizable moments without LRO already leads to an AHE, and that proof of ferromagnetic LRO requires additional experimental evidence.

**Figure 1:**
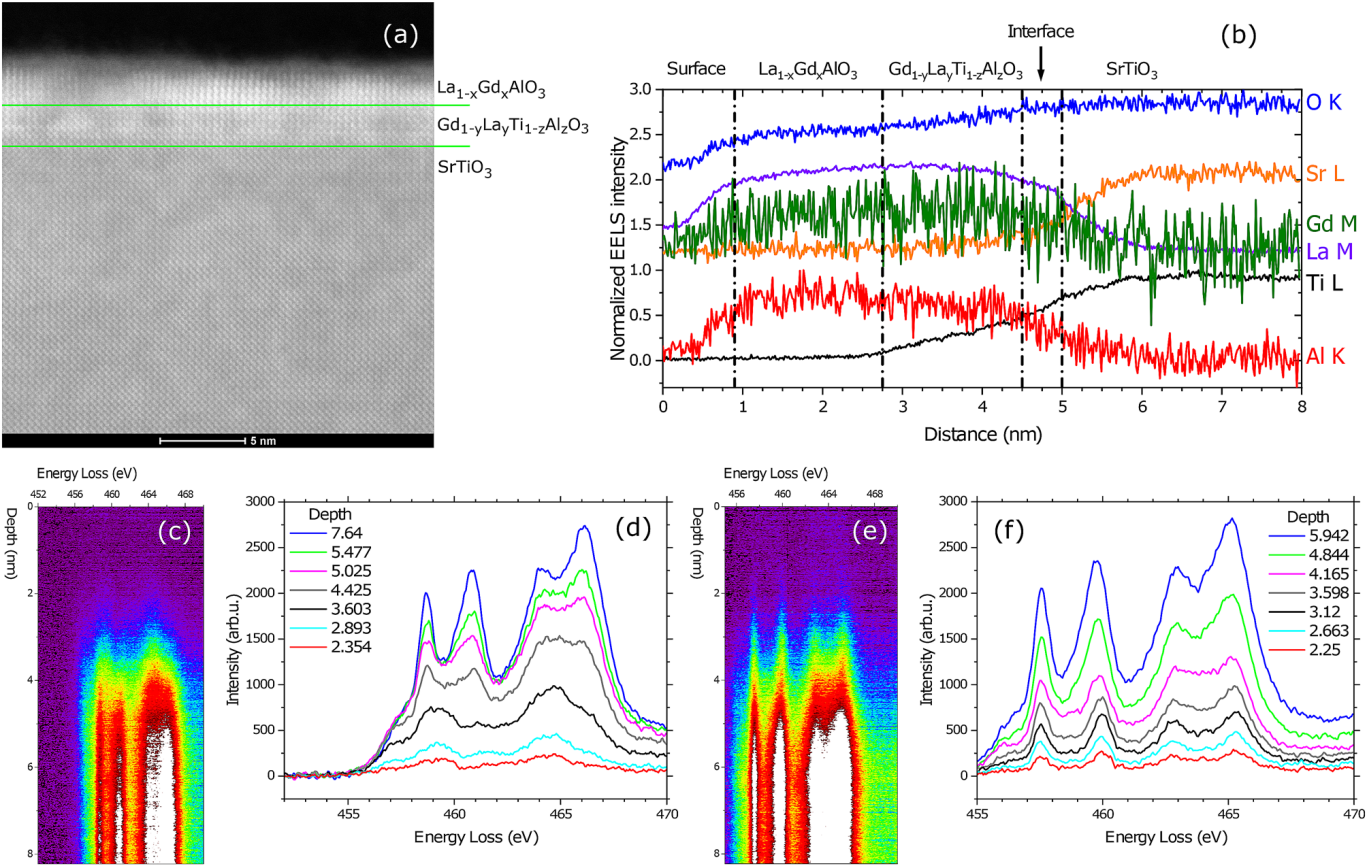
**(a)** TEM image of crystal structure at and close to the interface. The green lines show the position of the interface with STO, mainly from the Sr EELS data; and the presumed interface between GTO and LAO, taken from the Ti EELS data. **(b)** EELS analysis of spatial distribution of the various elements. **(c)** Ti L edge and **(d)** corresponding spectra in region with presence of Ti^3+^ in GLTAO layer. **(e)** Ti L edge and **(f)** corresponding spectra in region without of Ti^3+^ in GLTAO layer.

For our study we use delta doping by GdTiO_3_ (GTO) which is a ferrimagnetic Mott insulator^10^. In stoichiometric GTO the oxidation state of Ti is Ti^3+^. Therefore, GTO is polar, and the 2DEL will be formed at the GTO/STO interface. Interface conductance was indeed found in this system when grown by molecular beam epitaxy^11,12^. The research also demonstrated signatures of magnetism such as hysteretic behaviour of the magnetoresistance and anisotropic magnetoresistance^11,12^. AHE was not detected, probably due to the relatively low magnetic fields^12^. We have grown heterostructures with ultrathin GTO layers, in particular LAO(8)/GTO(2)/STO, with the numeral denoting the number of unit cells. The capping layer is essential to prevent overoxidation of the surface of rare earth titanates, uncapped samples tend to form magnetic dead layers with a higher content of Ti^4+^ instead of Ti^3+^^13–15^. Characterization by electron microscopy showed intermixing of La and Gd, leading to a structure La_1-x_Gd_x_AlO_3_/Gd_1-y_La_y_Ti_z_Al_1-z_O_3_/STO (LGAO/GLTAO/STO). Still, this serves our purpose, as it constitutes a system where magnetic ions are placed at, or close to, the conducting interface. In that sense, our current work is more similar to the work on LAMO cited above. In the following, we show that the 2DES in this system does not show ferromagnetism, but that at low temperatures, where Rashba spin-splitting is essential, it exhibits gate-tunable AHE. In particular, we find that the AHE at 3 K develops around the gate voltage where the system passes through the Lifshitz transition. That voltage will depend on the ’training’ of the sample, meaning to the maximum applied voltage in a given measurement cycle, due to the change of the potential well and the ensuing carrier trapping, which is a well known effect in LAO/STO^16–18^.

## Results

### TEM characterisation

Analysis by Scanning Transmission Electron Microscopy (STEM) revealed that the films are crystalline (Fig. 1a). At the same time Electron Energy Loss Spectroscopy (EELS) analysis indicated a severe intermixing in the sample (Fig. 1b). In particular strong interdiffusion of Gd, La and Al is present over the whole thickness of the deposited layers, turning them into La_1−x_Gd_x_AlO_3_ and Gd_1-y_La_y_Ti_z_Al_1−z_O_3_ instead of LAO and GTO respectively. Sr diffuses about 1 nm into the film whereas Ti diffuses further (around 2 nm) (Fig. 1b) yielding a thickness of GLTAO layer of about 5 u.c. Further investigation of the structure of the Gd_1-y_La_y_Ti_z_Al_1-z_O_3_ layer showed a varying amount of Ti^3+^ and Ti^4+^ along the film, as revealed by the study of the fine structure of the Ti L edges shown in Fig. 1c–f. In the first ‘GLTAO’ region shown in Fig. 1c,d, Ti is purely in the Ti^3+^ state (black, light blue and red spectra in Fig. 1d), whereas in the second ’GLTAO’ region in Fig. 1e,f the most of Ti is Ti^4+^. Data on the O K edge are shown in Supplementary Figure S1. Clearly, in spite of the capping with LAO, which should enhance the concentration of Ti^3+^, the growth of films in O_2_ atmosphere as well as the choice of STO as a substrate increases the concentration of Ti^4+^ in the (RE)TiO_3_ (RE = Rare Earth) layer^14,15^. Although the stoichiometric gradient in our films was not intentional, we stress again that transport properties presented in the rest of the Results section are reproducible in the control sample. Such robustness is understandable because the conducting media is a single crystal STO substrate, which properties govern most of the heterostructure physics.

**Figure 2:**
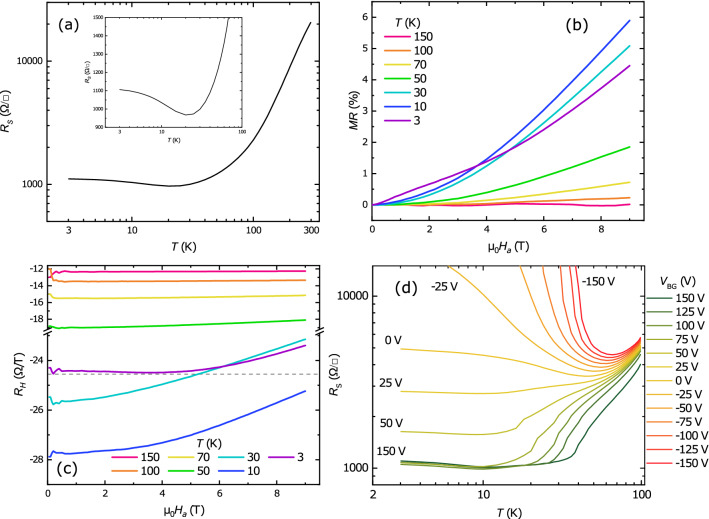
**(a)** Temperature dependence of the sheet resistance $R_S$ of the LGAO/GLTAO/STO sample during cool down. The same data with linear Sheet resistance scale in lower temperature region. **(b)** Field dependence of the magnetoresistance *MR* and **(c)** field dependence of the Hall coefficient $R_H$. The horizontal grey dashed line serves to highlight the nonlinearity of the Hall coefficient at 3 K. Panels **(a–c)** show data before applying a gate voltage. **(d)** Sheet resistance $R_S$ versus temperature at different back gate voltages $V_{BG}$ in the range from 150  to −150 V as indicated.

**Figure 3:**
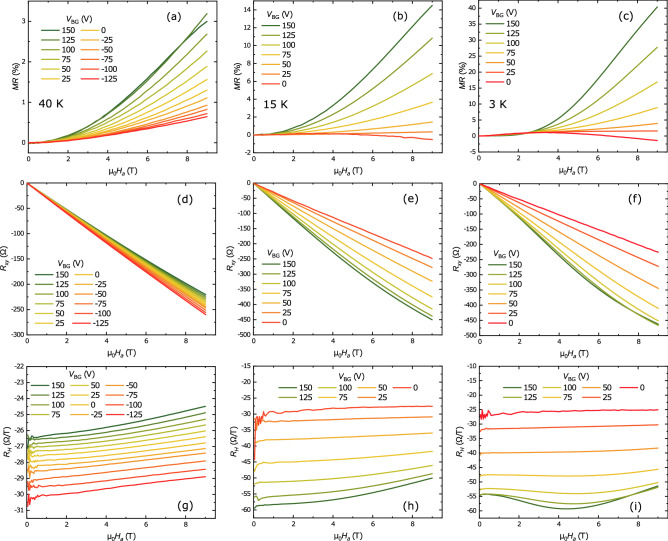
Magnetoresistance *MR*, Hall resistance $R_{xy}$ and Hall coefficient $R_H$ of the LGAO/GLTAO/STO sample for different positive back gate voltages $V_{BG}$ as indicated at the temperatures of 40 K **(a,d,g)**, 15 K **(b,e,h)** and 3 K **(c,f,i)**.

### Basic transport properties

LGAO/GLTAO/STO is conducting and exhibit temperature dependence of sheet resistance ($R_S$), which is comparable to LAO/STO^19^ (Fig. 2a). Also the magnetoresistance *MR* and the Hall resistance $R_{xy}$ were measured during cooldown. The magnetoresistance (*MR*) was calculated as:

1 $$\begin{aligned} MR = \frac{R_S(B)-R_S(0)}{R_S(0)} \cdot 100\% . \end{aligned}$$

As shown in Fig. 2b, the *MR* changes shape from almost flat to parabolic with decreasing temperature. At 3 K we note a different shape with a dip around zero field, which indicates the appearance of Weak Anti-localization (WAL), similar to what was shown earlier for LAO/ETO/STO^20^ and LAO/STO^2,21^. The Hall coefficient $R_H$ was extracted by dividing the Hall resistance by the applied field, $R_H = R_{xy} / (\mu _0 H_a)$. As shown in Fig. 2c, $R_H$ starts to deviate from flat behaviour (meaning a Hall resistance linear in the applied field) below 70 K. Such non-linear behaviour signals the presence of two bands, with low mobility 3d_xy_and highly mobile 3d_xz/yz_ carriers, respectively^3,22^. In other words, the system is above the Lifshitz point. At 3 K, a second non-linearity occurs at lower fields, in which the slightly parabolic shape around zero field becomes inverted. Such behaviour has already been observed in other STO-based heterostructures and was identified as a signature of AHE^3,6,23^. All in all, the basic transport characteristics show a behaviour which is quite typical for that of the LAO/STO family.

### Gate-induced AHE

Next, we studied the behaviour of the LGAO/GLTAO/STO sample upon applying a back gate voltage $V_{BG}$. Gating results in a tunable Metal-Insulator Transition (MIT), as shown in Fig. 2d. The $R_S(T)$ curves were measured by cooling down from 100 K at constant applied gate voltage. The change of the gate voltage, going down from 150 V, was always performed at 3 K. Note that, after applying a gate voltage, the sample has irreversibly changed because of the change in the shape of, and the number of carriers in, the potential well^17,18^. Zero volt data in this section therefore differ from zero volt data in the previous section. In particular, as we will see, at zero volt, the system is now below the Lifshitz point.

Below −25 V, the system becomes insulating at low temperatures. We do not observe saturation of $R_S$ in our samples, similar to delta-doped samples with $\mathrm{SrTi}_{1-x}\mathrm{Mn}_{\mathrm{x}}\mathrm{O}_{3}$^24^ but in contrast to such materials as ETO and LaCrO_3_, where a Kondo-like effect with saturation at low temperatures^25,26^ was observed. Furthermore, the system shows incipient Weak Localization (WL) behaviour (see below), which also has been observed in LAO/STO^2,21,27^. In the range from −25 V to 0, a pronounced minimum appears as function of temperature. For positive gate voltages, a small upturn in $R_S$ can be observed. Ref.^23^ indicated a correlation of a similar upturn and the emergence of AHE in a similar 2DES system, NdGaO_3_/STO (NGO/STO). At the same time, the MR changes shape from incipient WL to WAL, which disappears at high positive $V_{BG}$. All data confirm that the behaviour of LGAO/GLTAO/STO follows the scenario well known for LAO/STO, with the presence of a localized phase at negative $V_{BG}$, and a crossover to a conducting phase at positive $V_{BG}$, ascribed to a strong change in (Rashba) spin-orbit interaction by Caviglia et al.^2^.

To better understand the transport behaviour at low temperatures, and in particular at the lowest temperature of 3 K, we studied the evolution of the magnetotransport properties as function of back gate voltage more closely at three different temperatures, 40 K, 15 K and 3 K. All gate voltage sweeps were made by sweeping from 150 V to downward, ending at 0 V for 15 K and 3 K because of the MI transition. Comparing the results shows significant changes occurring when going to the lowest temperature. At 40 K the *MR* is small and has a parabolic shape in the whole range of gate voltages (Fig. 3a). At 15 K (Fig. 3b) and 3 K (Fig. 3c) the shape, in particular at low $V_{BG}$ diverges from parabolic, and a negative *MR* appears in high field. The (negative) Hall resistance at 40 K decreases with decreasing gate voltage, but increases at 15 K and 3 K (Fig. 3d–f). The Hall coefficient $R_H$ shows that the Hall effect is non-linear in the whole range of voltages at 40 K (Fig. 3g). At 15 K it becomes nonlinear above 25 V and at 3 K above 50 V (Fig. 3h,i). As was mentioned above, this nonlinearity of the Hall effect in high fields indicates the presence of a second band, in this case of 3d_xz/yz_ carriers, and its appearance signals that the system passes through the Lifshitz point. This is of obvious importance for the extraction of carrier concentrations and mobilities^3,22^.

### Extracting the anomalous Hall effect

Firstly, we calculate the longitudinal $G_{xx}$ and transverse $G_{xy}$ conductance from the relationship between resistance and conductance.

2 $$\begin{aligned} G_{xx}(B)= \frac{R_{S}(B)}{R_{S}^2(B)+R_{xy}^2(B)}, \quad G_{xy}(B)= \frac{-R_{xy}(B)}{R_{S}^2(B)+R_{xy}^2(B)}, \end{aligned}$$

**Figure 4:**
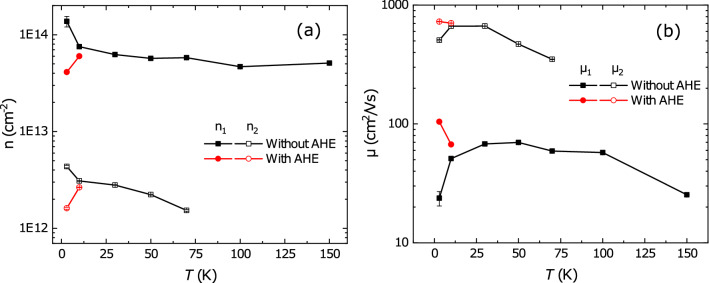
**(a)** Carrier concentration *n* and **(b)** mobility $\mu$ versus temperature for the LGAO/GLTAO/STO sample before applying the gate voltage. The black squares follow from the analysis without taking an AHE contribution into account, the red circles are the result of incorporating an AHE contribution as described in the text.

Then, to extract carrier concentrations and mobilities, we fit both of them using a two-band model.

3 $$\begin{aligned} G_{xx}(B)= \frac{en_1\mu _1}{1+\mu _1^2B^2}+\frac{en_2\mu _2}{1+\mu _2^2B^2},  G_{xy}(B)= \frac{en_1\mu _1^2B}{1+\mu _1^2B^2}+\frac{en_2\mu _2^2B}{1+\mu _2^2B^2} \end{aligned}$$

where *e* is the elementary charge. The temperature dependencies of both quantities are shown in Fig. 4 and yields the usual picture. We find a high-mobility band with low carrier concentration (of order $3 \times 10^{12}$ cm$^{-2}$) and a low-mobility band with high carrier concentration (of order $8 \times 10^{13}$ cm$^{-2}$). The data at 3 K show sharp changes in all values, however, because the model does not capture the ’magnetic’ contribution of the AHE to the Hall data $R_{xy}^{AHE}$. Gunkel et al.^23^ used the following phenomenological description to describe $R_{xy}^{AHE}$:

4 $$\begin{aligned} R_{xy}^{AHE}(B)= R_{0}^{AHE} tanh\left( \frac{B}{B_c}\right) . \end{aligned}$$

Here $R_{0}^{AHE}$ is the anomalous Hall coefficient. The description is based on the Langevin function for the behaviour of paramagnetic clusters, $\mathrm{B}_{\mathrm{c}}$ is a fitting parameter taking over the role of temperature in setting the energy scale for the field.

In a slightly different approach, Maryenko et al.^9^ used a Brillouin function to describe the non-hysteretic Anomalous Hall effect in a non-magnetic 2DEG in the very different system MgZnO/ZnO. For magnetic entities with $g=2$ and $J=1/2$, their description comes down to

5 $$\begin{aligned} R_{xy}^{AHE}(B)=R_{0}^{AHE} tanh\left( \frac{M_{eff}\mu _B B}{k_B T}\right) , \end{aligned}$$

with $M_{eff}$ and $R_{0}^{AHE}$ both fitting parameters.

Gunkel et al.^23^ used Eq. (4) to directly fit Eq. (3) for $R_S(B)$ and $R_{xy}(B)$ +$R_{xy}^{AHE}(B)$. The disadvantage of that method is the a priori assumption about the field-dependence of $R_{xy}^{AHE}$. We take a different approach in order to extract the carrier concentrations at 3 K. The idea is that in a small range of temperatures the mobilities and concentrations would not change abruptly or too much, and it will be possible to find the AHE by subtracting the Hall resistance curve at higher temperature without AHE from the Hall resistance curve at lower temperature with AHE.

**Figure 5:**
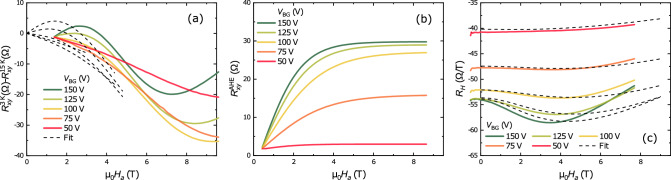
**(a)** Subtraction of the curves measured at 15 K and $V_{BG}$ as indicated from curves measured at 3 K (solid lines); and the fit with Eq. (6) (dashed lines). **(b)** Resulting AHE field dependence obtained from the fit (see text). **(c)** Measured Hall coefficients $R_H$ at 3 K and for different gate voltages $V_{BG}$ (solid lines) and the fit (dash lines).

Figure 5a shows the result $\Delta R(B)$ of subtracting $R_{xy}$(15 K) from $R_{xy}(3 K)$ for different back gate voltages. The AHE contribution is clearly visible as a wiggle on top of a negative slope. In an interval up to 3-6 T, this can be fitted quite well with Eq. (5) by subtracting the slope in the form of a linear residual correction:

6 $$\begin{aligned} \Delta R(B)=R_{0}^{AHE} tanh\left( \frac{M_{eff}\mu _B B}{k_B T}\right) +a \; B, \end{aligned}$$

where *a* is the slope of the linear correction and a fitting parameter, along with $R_{0}^{AHE}$ and $M_{eff}$. The value of *a* is around 5 - 10 $\Omega /T$, significantly less than $R_H$ itself, which is around 50 $\Omega /T$ at 3 K. The presence of the correction shows that the assumption of ’no change’ is not exactly valid. Supplementary Figure S2 shows the result of subtraction of curves at 40 and 15 K at different gate voltages. In that case only a high field non-linearity is present. Fig. 5b shows the curves as they came out from fitting Eq. 6, between 0 T and 3–5 T.

The analysis yields the gate dependence of the AHE at 3 K, but we can use the same approach to make a correction to the calculated carrier concentrations and mobilities of the sample before gating, given in Fig. 4. For this, we subtracted $R_{xy}$(30 K) from $R_{xy}(3 K)$ and $R_{xy}(10 K)$, and fitted the resulting curves with Eq. (6). Curves and fits are given in Supplementary Figure S3. We used the result to correct $R_{xy}$ at 10 K and 3 K for this AHE contribution and obtain *n* and $\mu$ with help of Eq. (3).

**Figure 6:**
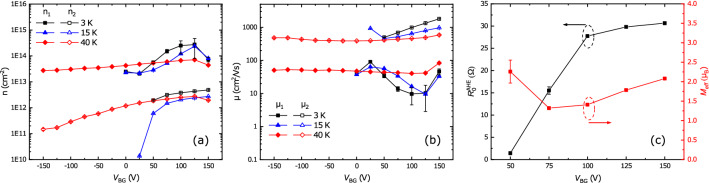
**(a)** Carrier concentration, and **(b)** mobility as function of gate voltage for different temperatures. **(c)** Anomalous Hall coefficient $R_{0}^{AHE}$ (black) and magnetic moment per atom $\mu _B$ (red) versus back gate voltage at 3 K.

The procedure removes the sharp increase of carrier concentrations and decrease of mobilities at 3 K (Fig. 4) and actually seems to overcompensate the AHE correction somewhat. A closer look at the Hall coefficient (Fig. 5c), which is a more sensitive parameter than $R_{xy}$ itself, reveals that the method works better at low fields, explaining the overcompensation since the two-band behaviour is a high field phenomenon. Not being able to fit the whole field range probably arises from the use of the empirical function proposed by Gunkel et al.^23^ and leads to a limited precision of extracted carrier concentrations and mobilities. However, in the absence of a theory for AHE, it is hard to find appropriate form of the AHE term in the conductance representation. The resulting fitting parameters $n, \mu , R_{0}^{AHE}$ and $M_{eff}$ are presented in Fig. 6.

We can note a few things. At 40 K a value for the carrier concentration of the second band is found for all gate voltages (Fig. 6a). For 15 K and 3 K, those carriers are only found for positive gate voltage. Carriers from the first band show a significant increase in concentration and a significant decrease in mobility upon gating at 15 and 3 K (Fig. 6a,b). Also, it is clear from the results, and emphasized in Fig.6c that the AHE is only observed close to or after the Fermi level crossing the Lifshitz point, when the second band comes into play. This is one of the main results of the paper, to be discussed below.

### The question of ferromagnetism

Summarizing the zero-volt and gated data, in the virgin sample we found two-band behaviour below 70 K, and signatures for the AHE at 3 K. After gating, the Lifshitz point shifted to a gate voltage of roughly 50 V. Above this value, in the two-band regime, we again find the AHE, in agreement with previous results on LAO/ETO/STO^6^. However, we did not observe AHE at 15 K and 40 K in spite of the clear presence of the second type of carriers (Fig. 3g,h). At these temperatures signatures of WAL were completely absent, indicating a possible important role for SOC in observation of AHE. Although the onset and increase of the AHE with increasing gate voltages is accompanied by the disappearance of WAL (Fig. 3c), Stornaiuolo and co-workers argued that the appearing of AHE may mask spin-orbit coupling rather than suppress it^20^. Moreover, with increasing gate voltage the carrier concentration is also increasing, leading to a stronger contribution of orbital effects in out of plane MR.

The occurrence of AHE is often taken as a signature of ferromagnetism, but we were not able to detect any hysteresis in our magnetotransport measurements down to 3 K. The same behaviour was reported for an interface between paramagnetic NGO and STO^23^. The occurrence of AHE was explained with the polarization of magnetic moments, more specifically by the rotation of moments around the out-of-plane hard axis in a magnetic field perpendicular to the sample surface^28^. In order to investigate this further, we performed measurements with a scanning SQUID microscope^29^ on non-gated samples and samples gated at 150 V, 0 V and -150 V. The resulting scans are presented in Supplementary Figure S4. They did not show any signatures of ferromagnetic domains at 4.2 K, nor did they show ferromagnetic patches such as observed by scanning SQUID in LAO/STO structures^30–32^. This could be due to the fact that domains are smaller than our resolution. At the same time, however, EELS data indicated the presence of regions with Ti^3+^and regions without it, and such a distribution of Ti^3+^with a more uniform distribution of Gd can lead to superparamagnetic behaviour. The measurement setup did not allow to apply a magnetic field and gate voltage simultaneously so we cannot completely exclude a scenario of superparamagnetic rather than paramagnetic behaviour, in which larger ferromagnetic domains form in an external magnetic field. Also, the absence of a change in the ferromagnetic landscape when applying a gate voltage is consistent with study on LAO/STO^30^.

## Discussion

We have shown in the previous sections that, in particular at 3 K, a non-hysteretic AHE contribution to the Hall resistance can be found above a certain gate voltage. Here we discuss its possible origins. Naively, the answer could be that ferromagnetism is induced by the insertion of ferrimagnetic GTO and gate-doping enough carriers to have sufficient exchange interaction. However, we were not able to detect ferromagnetic regions by scanning SQUID microscopy. Taking together the results of EELS and scanning SQUID, we conclude that our GLTAO layer is rather in a paramagnetic or superparamagnetic state than ferrimagnetic, due to strong intermixing.

Strictly speaking, AHE need not be signature of ferromagnetic order. A growing number of reports, both theoretical and experimental, shows that it can be seen in other systems, for example in paramagnets^33–37^, including the already mentioned system MgZnO/MgO^9^, in superparamagnets^38–40^, in antiferromagnets^41,42^ and recently in LaAlO_3_/SrTiO_3_ through magnetism at ferroelastic domain walls in the STO^43^. In our case, the AHE increases with gate voltage and therefore appears to be connected to the transition through the Lifshitz point and the onset of conductance through a second band. This allows for two new mechanisms to appear. One has to do with the magnetic interactions. The d_xy_ band is circular, lies in the plane of interface, and 3d_xy_ electrons are coupled antiferromagnetically to magnetic moments^44,45^. The d_xz/yz_ bands, on the other hand, have highly elliptical Fermi surfaces directed along crystal axes and the 3d_xz/yz_ electrons are thought to couple ferromagnetically to the local Ti$^{3+}$ magnetic moments^44,45^ and, in our system, to the Gd 4f magnetic moments as well. Ferromagnetic interactions therefore may appear beyond the Lifshitz point. The other and probably more important mechanism is the enhanced Rashba spin splitting occurring near the band crossing between the light and heavy bands^46,47^, which leads to amplified spin-orbit coupling^48,49^. Specifically, it has been shown that the characteristic spin-orbit fields can increase almost an order of magnitude with increasing gate voltage^1,2,50^. Against this picture pleads that the AHE in our system has been observed only at temperatures around or below 10 K, whereas the two band behaviour is generally observed in a much wider temperature range. First, it should be remembered that, if the system is (super)paramagnetic, the magnetization will decrease quickly with increasing temperature. Moreover, the physics here is more complicated than in the case of semiconductors. Diez et al.^48^, for instance, showed that, around the Lifshitz point, the density of states increases steeply with band energy which leads to a strong lowering of the chemical potential between about 5 K and 20 K, and a different distribution between filled and empty states available for scattering. Such effects should also affect the strength of SOC and the generation of the AHE. In paramagnetic semiconductors with linear Rashba SOC, the appearance of the AHE will be depend on the energy scales of magnetization and the product of spin-orbit coupling constant and Fermi wavevector. If the latter is much larger, the AHE will be absent due to cancellation of intrinsic and extrinsic terms^35^. Indeed, an increase of $R_{0}^{AHE}$ and a decrease of with $R_{S}$ indicates that the mechanism of gate control is complicated, and either multiple mechanisms are responsible for AHE either our samples have strong effects of the disorder. All in all, there are ample indications that the gate-induced onset of AHE at the lowest temperatures is due to the physics of the Lifshitz-point, while the disappearance of AHE at higher temperature, notwithstanding the presence of two bands, could be explained by the same physics. It is interesting to note that the system $\gamma$-Al$_2$O$_3$, or (GAO)/STO, shows one-band behaviour, no Lifshitz point, and quite different transport physics^43^. The proposed mechanism can also give a new insight in the AHE dependence on oxygen pressure reported by Ref.^23^. In that work, the authors proposed that magnetism is controlled (indirectly) by Sr vacancies and not by oxygen vacancies. High growth pressure leads to enhancement of the amount of Sr vacancies, and of AHE, but to a smaller number of oxygen vacancies, and consequently to a smaller concentration of localized Ti^3+^ moments. At the same time, oxygen vacancies do not just control magnetism, they determine the electrostatic boundary conditions of the quantum well^51^ and thus the band filling and the strength of the SOC, which is essential for the AHE as has been discussed above.

## Methods

Most of the previous studies on GTO heterostructures were performed on the films grown by Molecular Beam Epitaxy (see for example Ref.^52–54^) and only few by Pulsed Laser Deposition (PLD)^55,56^. In this work we grow samples on $\mathrm{TiO}_{2}$-terminated STO by PLD from an oxygen rich target GdTiO_3+x_ at 850 $\circ ^{\rm{C}}$ at $1 \times 10^{-4}$ mbar O_2_ nominal pressure. The repetition rate and laser fluency were set at 1 Hz and 1.3 J/cm^2^, respectively. The samples were cooled down to room temperature at the growth pressure. The growth of the films was monitored by Reflection High-Energy Electron Diffraction (RHEED), which also yielded an estimate for the film thickness. Nominal layer thicknesses were chosen as 8 unit cell (u.c.) for LAO and  2–2.5 u.c. for GTO. As will be shown later the real thicknesses and compositions of layers were different, due to strong intermixing. Also, the growth of a GTO layer with a reliable thickness turned out to be a challenging task due to the sensitivity to the growth conditions. In particular, rare earth titanates have a tendency to form a pyrochlore phase Re_2_Ti_2_O_7_ in an oxygen-rich environment^15,54^. RHEED oscillations were hardly pronounced at the pressure we used, although the RHEED patterns did exhibit 2D growth (See Supplementary Figure S5). At the same time, lowering of the O_2_ pressure would lead to enhancement of oxygen vacancies in STO and, therefore, to the bulk conductance in STO^57^.

Magnetotransport properties were measured using an automated measurement platform (a PPMS from Quantum Design) with a home built electrical insert to be able to gate the samples. They were measured in the van der Pauw geometry^58,59^ at temperatures down to 3 K and magnetic fields up to 9 T. All measured field dependencies were (anti-)symmetrised. An example of non-symmetrised data is shown in Supplementary Figure S6. To study magnetism, scanning SQUID microscopy measurements were performed at 4.2 K without external magnetic field. The spatial resolution 10 $\mu m$ and the noise floor is $\approx$ 50 nT. A control sample showed the same qualitative behaviour as the results reported here. A second control sample was cut in two, with one part being used for analysis of the structure and chemical composition by STEM and EELS. The other part was used for transport measurements and showed results which were consistent with the earlier two samples.

## Supplementary Information

Supplementary Figures.
