# Supplementary material for: Gate-tuned anomalous Hall effect driven by Rashba splitting in intermixed LaAlO3/GdTiO3/SrTiO3
Source: Sci Rep. 2021 May 21;11:10726. doi: 10.1038/s41598-021-89767-3 (PMC8140084; doi:10.1038/s41598-021-89767-3)
Supplement: Supplementary file 1 — Supplementary Figures. [file 41598_2021_89767_MOESM1_ESM.pdf]

Supplementary Information for Gate-tuned Anomalous Hall Effect Driven by Rasba Splitting in Intermixed $LaAlO_3/GdTiO_3/SrTiO_3$

N. Lebedev,[1] M. Stehno,[2] A. Rana,[3] P. Reith,[4] N. Gauquelin,[5]
J. Verbeeck,[5] H. Hilgenkamp,[4] A. Brinkman,[4] and J. Aarts[1]

[1]*Kamerlingh Onnes Laboratory, Leiden University,*
P.O. Box 9504, 2300 RA Leiden, The Netherlands

[2]*Physikalisches Institut (EP 3), Universität Würzburg,*
*Am Hubland 97074 Würzburg, Germany**

[3]*School of Engineering and Technology,*
BML Munjal University (Hero Group), Gurgaon, India - 122413

[4]*MESA+ Institute for Nanotechnology,*
University of Twente, P.O. Box 217,
7500 AE Enschede, The Netherlands

[5]*Electron Microscopy for Materials Science, University of Antwerp,*
Campus Groenenborger Groenenborgerlaan 171, 2020 Antwerpen, Belgium

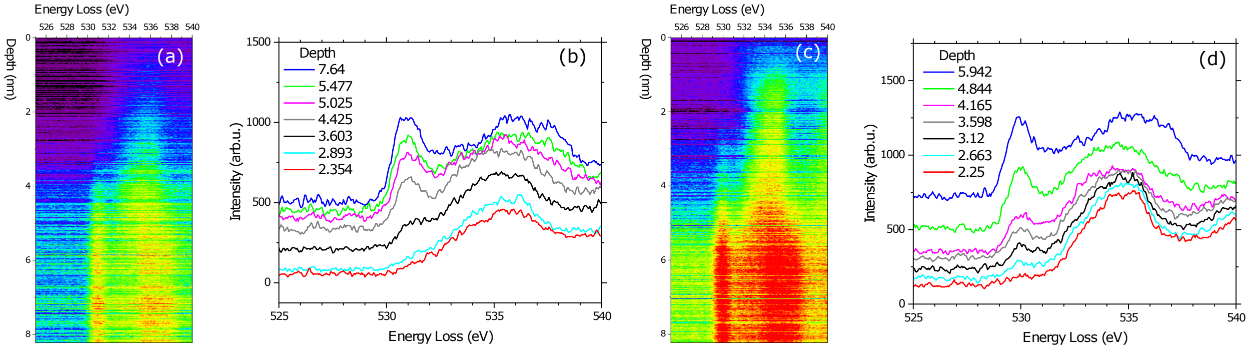

FIG. S1. EELS data; (a) O K-edge signal as function of distance from the interface in a region where Ti^{3+} is present in the GLTAO layer and (b) corresponding spectra in that region. (c) O K edge in a region where Ti^{3+} is absent in the GLTAO layer and (d) corresponding spectra in that region.

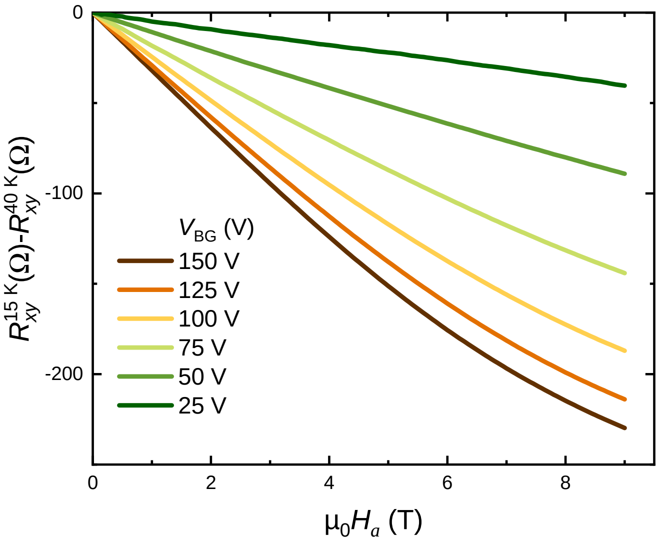

FIG. S2. The result of substraction of Hall resistances R_{xy}^{40K} measured at 40 K from curves measured at 15 K, at different gate voltages as indicated.

---

* The first two authors contributed equally

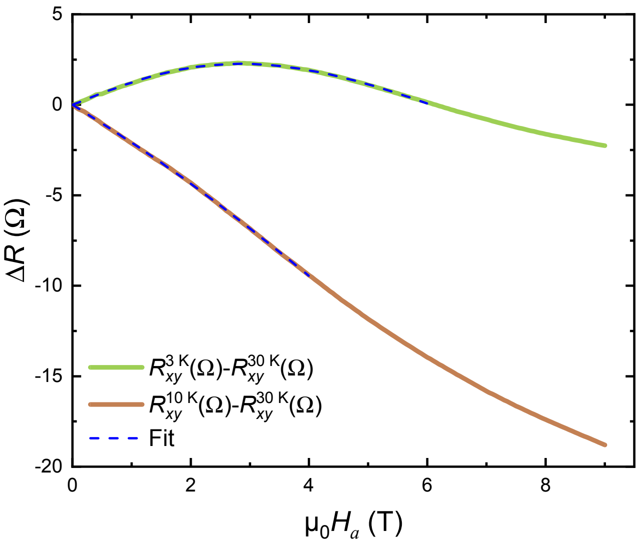

FIG. S3. (a) Result of the substraction of the Hall resistance R_{xy}^{30K} measured at 10 K from the curves measured at 3 and 10 K before applying a back gate voltage V_{BG}. The results of the fit are $R_0^{AHE} = 9.28\pm0.08$ and $M_{eff} = 1.33\pm0.01$ at 3 K and $R_0^{AHE} = 1.78\pm0.15$ and $M_{eff} = 6.03\pm0.29$ at 10 K

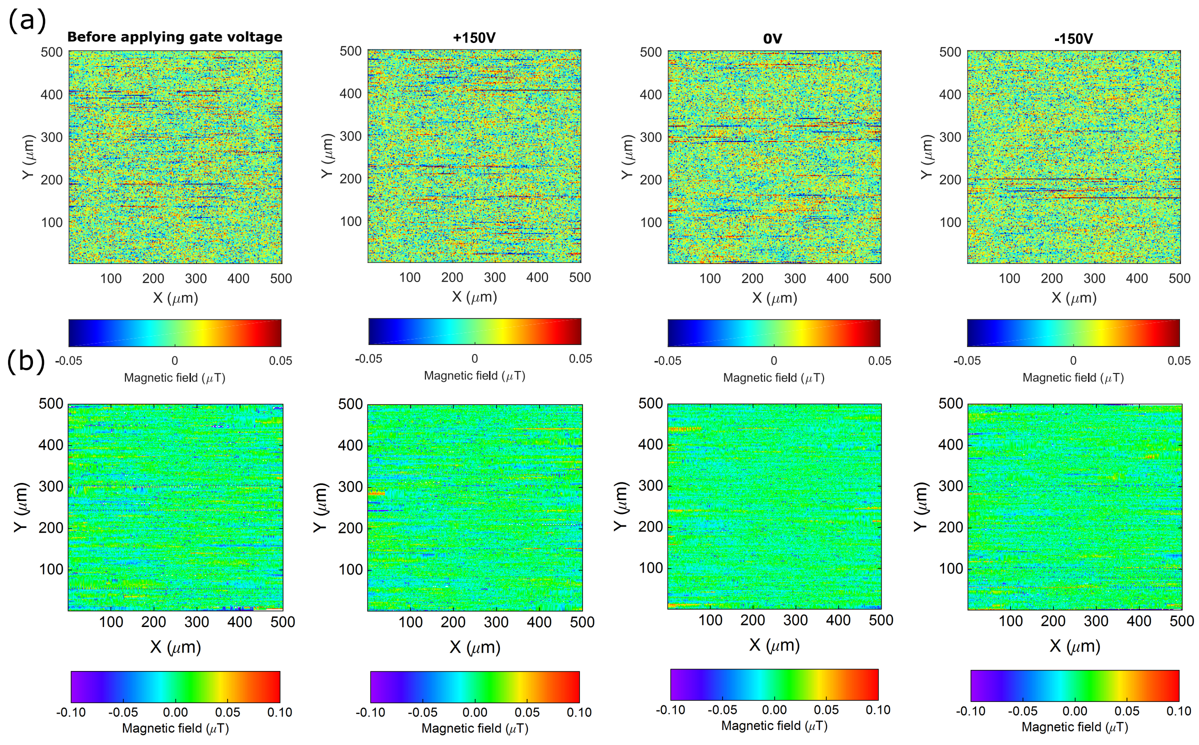

FIG. S4. Scanning SQUID experiments on (a) the sample reported in the main text and (b) a reference sample.

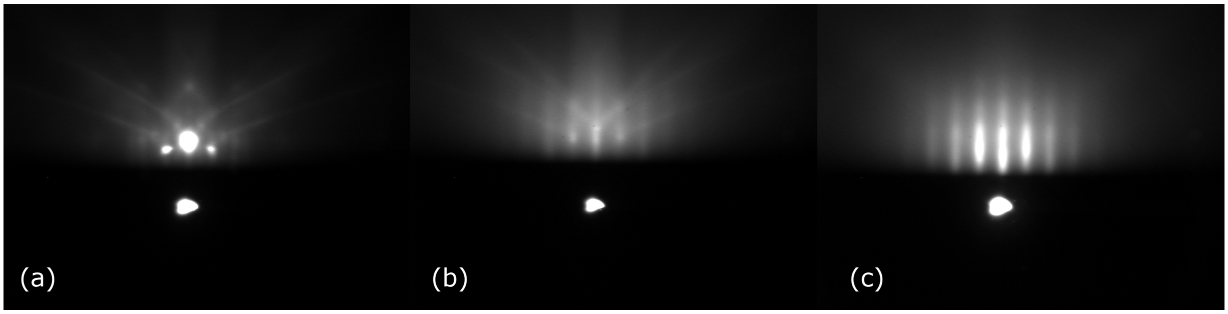

FIG. S5. RHEED patterns (a) before deposition at room temperature, (b) after deposition of $GdTiO_3$ and (c) after deposition of $LaAlO_3$ at 850°C.

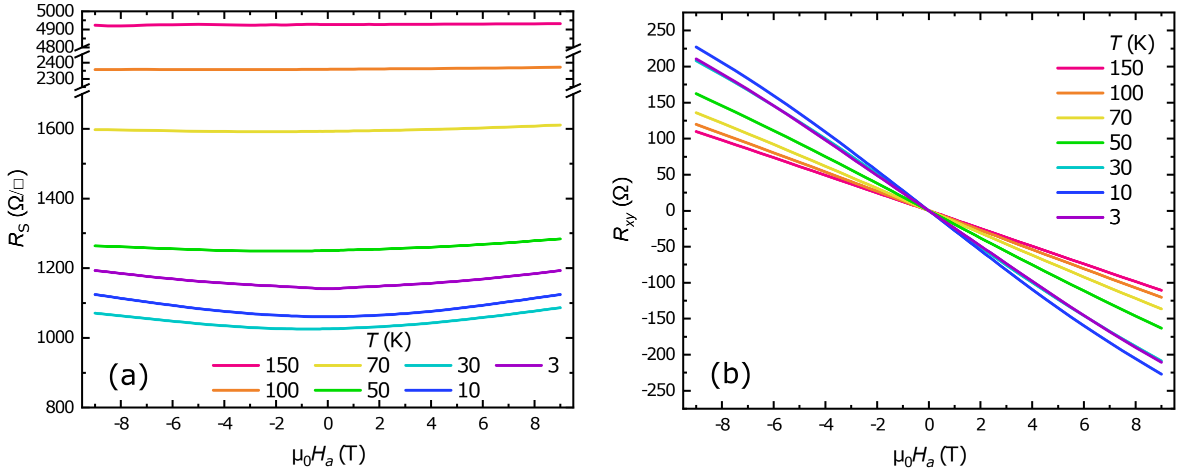

FIG. S6. (a) Non-symmetrized field dependence of the Sheet resistance R_S (calculated as $\frac{\pi}{ln2}\frac{R^{vertical}+R^{horizontal}}{2}$ and not via van der Pauw equation as in the main text) and (b) non-antisymmetrised field dependence of the Hall resistance R_{xy} at different temperatures. We used measured voltages in different van der Pauw configurations to obtain $R^{vertical}, R^{horizontal}, R_{xy}$.
